# Mapping the knowledge domains of medical textiles: A review

**DOI:** 10.1097/MD.0000000000035956

**Published:** 2023-11-10

**Authors:** Zhiqun Liu, Fangping Yin, Nan Ruan, Zongzhan Gao

**Affiliations:** a Library, Xi’an Polytechnic University, Xi’an, China; b School of Mechanics, Civil Engineering and Architecture, Northwestern Polytechnical University, Xi’an, China.

**Keywords:** bibliometric analysis, mapping knowledge domains, medical textiles, medical textiles material

## Abstract

As the world’s textile industry shifts towards manufacturing high value-added textile structures and products, medical textiles have drawn extensive attention from researchers and the related research field is rapidly developing in recent years. To provide readers a systematic overview of this research field, a comprehensive bibliometric analysis of scientific publications related to the field in performed and visually presented using the software CiteSpace and VOSviewer in this paper. Totally 2839 papers have been retrieved and collected from the core database of Web of Science™. First, the papers are divided into several groups and quantitatively analyzed based on the year of publication, the citations in each year, and the disciplines involved in the papers. VOSviewer is adopted to analyze the collaboration among countries, organizations, and authors in the research community as well as their research output and influence in terms of citation. Then the major journals in the field are identified through performing co-citation analysis on source journals of all references cited in the retrieved papers. In addition, the highly cited papers and their references are listed in this paper. They offer researchers a glimpse of the internal relationship of scientific literature and the dynamic structure of scientific evolution. Finally, the co-occurrence analysis of keywords is also performed using VOSviewer and CiteSpace. The connection between various disciplines in the research field is revealed, so that the scientific development history, the research hotspots, and main research directions in the field can be traced.

## 1. Introduction

High value-added textile structures and products like medical textiles, protective textiles, and smart textiles have become the development trends of the textile industry worldwide.^[1]^ Biomedical textiles, also known as medical textiles, are a kind of biomedical materials that are deeply integrated with textiles, materials, biology, medicine, and other related disciplines. These materials have structural advantages which cannot be matched by other kinds of biological materials.^[2]^ Medical textiles are textiles used in medical institutions for health care and medical treatment. According to their applications, medical textiles are divided into non-implantable, implantable, in vitro textiles and health care products. Medical textiles are divided into knitted fabrics, woven fabrics, and non-woven fabrics according to their structure.^[3]^ Medical textiles are used to make curtains and bedding in health care environments as well as wound dressings, bandages, and other products. Medical apparel products include coveralls, shoe covers, full body suits, gloves, separate sleeves, scrubs, surgical gowns, surgical masks, and scrub caps.^[4]^

An integrated part of the concept of textile biomedical engineering is that the role of textile devices in medical treatment is becoming more and more important with the advancement of science and technology. This is by means of drug delivery, regenerative medicine, tissue engineering and artificial implantation.^[5]^ The advanced medical textiles segment is growing significantly with major expansions in implantation devices, medical devices, bandages, and pressure clothing, wound healing, infection control, barrier materials, controlled release, sanitary products, new smart textile products and textronis.^[6]^ In recent years, medical textiles have drawn a lot of attention and developed rapidly in the field of textile.^[7]^ With the aging of the population and the on-going COVID-19 pandemic, the global demand for medical textiles will possibly increase in the future.

Recent published literatures mainly focus on the properties, applications,^[8,9]^ manufacturing technology^[10–15]^ turning process, and mechanical properties^[16]^ of medical textiles. There are few literatures that systematically reviews medical textiles from the perspective of bibliometrics. Bibliometrics is a research method that uses metadata such as titles, authors, and their affiliates, keywords, publication types and references of publications for quantitative analysis.^[17]^ Bibliometric data, including titles, authors, affiliations, journals, keywords, and references, can generate direct citation networks, co-occurrence networks, and bibliometric coupling networks for further analysis.^[18]^ Based on co-citation network, the software CiteSpace can be used to study the turning points, research frontiers and trends of a research area.^[19]^

In this study, totally 2839 scientific publications related to medical textiles have been retrieved and collected from the database of the Web of Science™. Using the software CiteSpace and VOSviewer, a bibliometric analysis is performed on the collected literature. The analysis result is presented, discussed, and summarized in the paper. The main findings of this paper reveal the collaboration between countries or regions, organizations, and authors in the research field. Furthermore, the knowledge base, the research hotspots, and the main research directions in the field are uncovered through generation and analysis of the keyword co-occurrence network, the literature co-citation network, and the keyword clusters, respectively. The comprehensive and quantitative review presented in this paper can help researchers understand the evolution, the state-of-the-art in the research field of medical textiles.

The paper is structured as follows. The data source and research tool are explained in the section “Data samples source and research tools,” which describes the source of data and how it is collected. The software CiteSpace and VOSviewer are also briefly introduced in this section. The results of bibliometric analysis are presented in the following sections. In the section “Yearly and citations distribution of articles,” the yearly publication and citations of the collected 2839 articles are analyzed. The research directions involved in the collected literature are summarized in section “The main research areas involved areas involved.” The software VOSviewer is used to analyze the collaboration between countries, organizations, authors, as well as their publications and citation. The analysis result is presented and discussed in sections “Analysis of co-authorship between countries or regions,” “Analysis of co-authorship between organizations,” and “Analysis of co-authorship between authors.” In section “Source journals analysis,” VOSviewer is used to analyze the total citations and to identify the major journals in the research field of medical textiles. The most cited papers in the field are found out and presented in section “Citation analysis of articles.” In section “References co-citation analysis,” the references cited by the collected papers are analyzed using VOSviewer. The 10 most co-cited references are identified and presented in this section. In section “Research hotspots,” the co-occurrence cluster analysis is performed on all keywords included in the collected papers using VOSviewer. Twenty most co-occurred keywords are listed in the section. Keywords with relatively high frequency of co-occurrence are clustered, and the possible research directions related to each cluster are discussed in this section, too. To reveal the evolution of research directions involved in each cluster through a timeline view, CiteSpace is additionally used to perform the co-occurrence cluster analysis of keywords, and the result including the burst detection keywords, high co-occurrence keywords, etc. is presented in section “Evolution of research directions and research fronts.” All analysis results, key findings, strengths, weaknesses, and limitations of this paper are summarized in section “Conclusions.”

## 2. Data samples source and research tools

The investigated literatures in this paper are retrieved from the core collection of Web of Science™, which is an important database for accessing to global academic information. It covers the fields of natural science, engineering technology, biomedicine, social science, arts, and humanities. The database includes more than 26,000 influential academic journals, more than 200,000 conference proceedings and more than 100,000 scientific and technological books worldwide. A complete record and citations of the investigated literatures including titles, authors, authors’ organizations, publication years, source journals are exported from Web of Science™.

The method used for collecting data is topic search. When an article contains any of the searched terms in its title, abstract or keywords, the article will be included in the collection. The formula TS = (medical textile* or medical textile* material or biomedical textile* or implantable textile* or healthcare and hygiene textile*) AND DT = (Article or Review or Early Access), Index Date = (1900-01-01 to 2023-09-25) is used to search in Science Citation Index Expanded (SCIE) and Social Sciences Citation Index (SSCI). Both SCIE and SSCI belong to the core collection of Web of Science™. The search time is September 26, 2023. Totally 2839 papers were retrieved, of which the publication time ranges from the year 1979 to 2023. These papers are published in 908 journals by 3006 research organizations and 11,146 authors from 101 countries or regions. Among them, there are 2269 articles, 570 reviews, and 55 early access, which account for 79.923%, 20.077%, and 1.937% of all retrieved papers, respectively.

In this paper, CiteSpace and VOSviewer are mainly used for bibliometric analysis of the collected literatures. CiteSpace is a popular software used by many researchers worldwide to analyze literature information. It is well developed for presentation of the structure and distribution of scientific knowledge for scientific metrology, data analysis. Information can be easily visualized by the software. It also facilitates the generation of different types of knowledge graphs and visual citations of the literature landscape.^[20]^ CiteSpace, developed by Professor Chaomei Chen, an internationally renowned expert in information visualization at Drexel University, USA,^[21]^ refers to a Java application to visually analyze literature and co-citation networks.^[22]^ Burst detection, mediated centrality, and heterogeneous networks regarding literate information can all be analyzed and displayed by CiteSpace. Based on the data derived from the literature, 3 main functions in CiteSpace can be used to perform visual analysis on literature. Such functions are detecting of the nature of specialized research frontiers, labeling and clustering specialized research areas, and identifying the research trends and abrupt changes. A valuable, timely, reproducible, and flexible method to track the development of research trends and to identify vital evidence can be provided by CiteSpace.^[23]^ VOSviewer is another useful visualization tool widely used by researchers all around the world to conduct bibliographic analysis.^[24]^ VOSviewer is based on Visualization of Similarities (VOS) technology, which has unique advantages in mapping knowledge domains, especially in the aspect of clustering.^[24]^ VOSviewer is also capable to construct and present bibliometric maps, in which links between scientific publications, scientific journals, researchers, research organizations, countries, keywords can be constructed in the forms of co-authorship, co-occurrence, citation, bibliographic coupling, and co-citation.^[24]^ The number of links and the total strength of those links can be evaluated for visualization of the network.^[25]^

**Figure 1.**
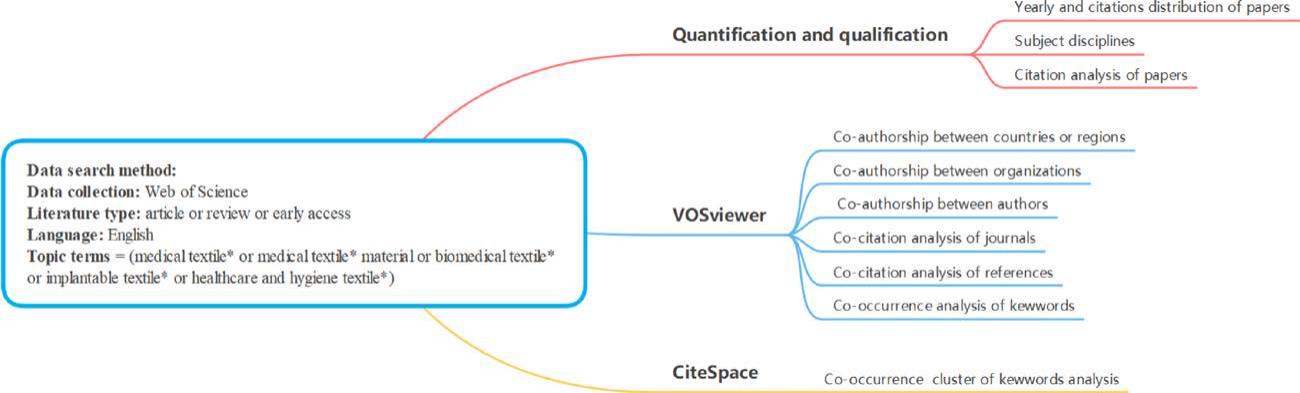
Flowchart of the analysis.

In addition to CiteSpace and VOSviewer, as can be seen from Figure 1, VOSviewer is used to analyze the distribution of research strength including the collaboration distribution of countries or regions and organizations, co-citation analysis of journals, co-citation analysis of references and co-occurrence analysis of keywords. CiteSpace is used to analyze the co-occurrence cluster of keywords to determine the evolution of research hotspots, research direction and research fronts.

## 3. Results and discussion

### 3.1. Yearly and citations distribution of articles

**Figure 2.**
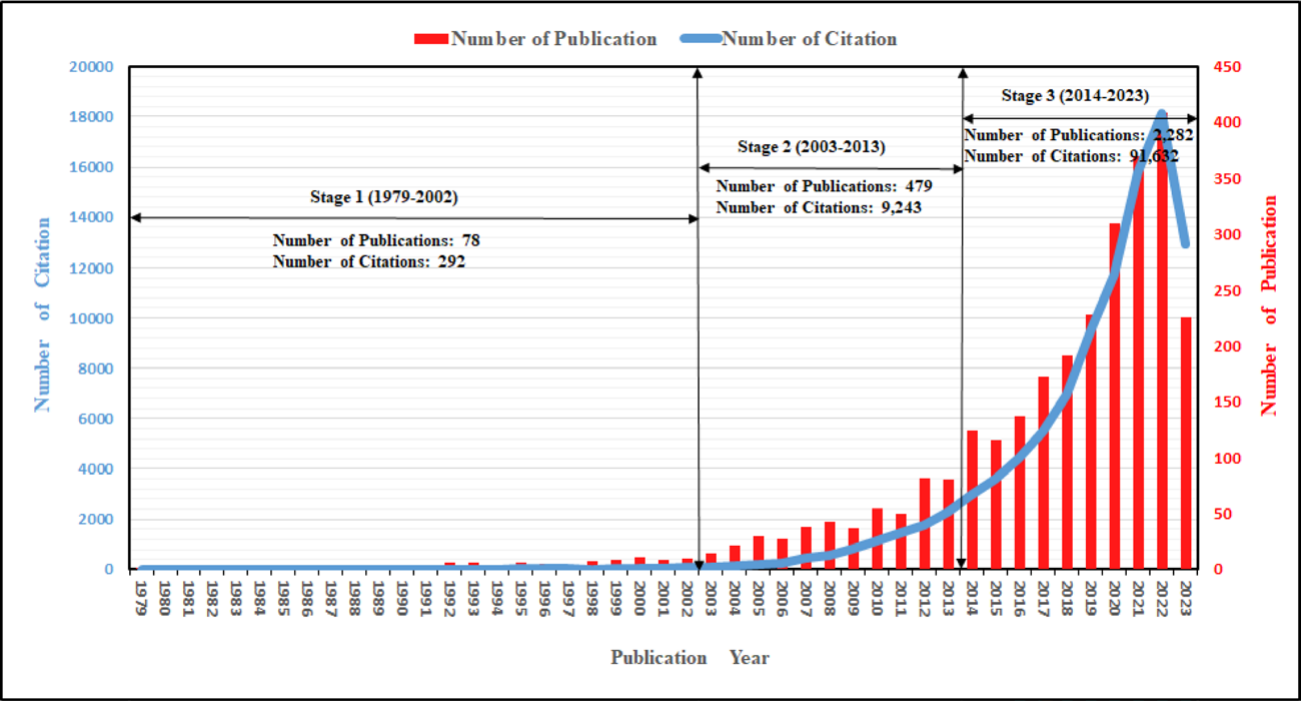
Yearly distribution of publications and citations related to medical textiles. Number of citation (blue); number of publication (red); and publication year (black).

Focusing on medical textiles and other related keywords, totally 2839 articles published between the year 1979 and 2023 are retrieved from the SCIE and SSCI database. These articles have been cited for 101,167 times, and this number remains 97,778 after removing self-citation. On average, each article is cited for 35.6 times with an h-index of 135. As shown in Figure 2, according to the number of publications and citations, the time frame from 1979 to 2023 can be roughly divided into 3 stages.

Stage 1 ranges from 1979 to 2002. This stage is a long bud stage in the research area of medical textiles. There are only 78 related published articles with 292 recorded citations during these 24 years. The most cited paper published at this stage is a review with title “Review of applications for advanced 3-dimensional fiber textile composites” by “Mouritz AP” published in “Composites Part A-Applied Science and Manufacturing” in 1999. This paper has been cited 839 times till September 29, 2023. It mainly discusses the usage of various textile processes such as braiding, weaving, stitching, and knitting of 3D fiber reinforced polymer composites with their current and future potential applications.^[26]^

Stage 2 covers the years between 2003 and 2013. This stage is a leap stage in the research area of medical textiles. During this period, 479 articles related to the research area are published, and the number of citations jumped to 9243. The most cited paper is a review titled “Nanosilver: A nanoproduct in medical application” by “Chen X.” published in “Toxicology Letters” in 2008, with a total number of citations of 1432 till September 29, 2023. This paper focuses on the medical use of nanosilver and related nanomaterials. Thanks to its strong antimicrobial effect, nanosilver coatings are used on various textiles as well as certain implants.^[10]^

Stage 3 starts from 2014 to 2023. This stage is exhibiting a rapid development in the research area related to medical textiles. Within only 9 years, already 2282 articles have been published and cited for 91,632 times. Of which the most cited one is an article titled “Biomimetic 4D printing” by “Gladman A. Sydney” published in “Nature Materials” in 2016. It is been cited 1868 time till September 29, 2023. This article focuses on biomimetic 4D printing of composite hydrogel structures with localized, anisotropic swelling behavior controlled by the alignment of cellulose fibrils along a defined 4D printing path.^[27]^

### 3.2. The main research areas involved

Many research areas are involved in medical textiles. The retrieved 2839 articles can be attributed to totally 105 research areas, of which the main areas are material science, engineering, chemistry. Table 1 lists the number of articles in each research area involved. Regarding the number of publications and citations, materials science is the most involve discipline, with 1219 published articles, followed by chemistry with 639 articles, and engineering with 629 articles, accounting for 42.938%, 22.508%, and 22.156% of all retrieved papers, respectively.

**Table 1:** Top 10 research areas distribution in medical textiles.

Research areas	Number of publications (1979–2023)	Percentage	Number of publications (1979–2013)	Number of publications (2014–2023)
Materials Science	1219	42.94	239	980
Chemistry	639	22.51	82	557
Engineering	629	22.16	98	531
Polymer Science	437	15.39	87	350
Science Technology Other Topics	343	12.08	25	318
Physics	324	11.41	43	281
Instruments Instrumentation	135	4.76	14	121
Biochemistry Molecular Biology	120	4.23	9	111
Biotechnology Applied Microbiology	103	3.63	23	80
Telecommunications	111	3.91	8	103

The table is divided into 2 columns according to the publication year of the articles. In the rapid development stage, the research areas involved are limited to materials science, engineering, and chemistry. The data in Table 1 are retrieved from the core collection of Web of Science™, and the identification of the research areas involved in the literature is not strictly limited.

### 3.3. Analysis of co-authorship between countries or regions

The collaboration between countries, regions, and organizations in the world is becoming increasingly frequent and close. “Research collaboration” can be defined as researchers doing research together to achieve the goal of common new scientific knowledge.^[28]^ In practice, scientific collaboration has many forms of expression and cooperation. Scientific collaboration refers to that different authors, organizations, countries, or regions contribute themselves to an article at the same time.^[29]^

In this paper, the co-authorship function of VOSviewer is used to analyze the collaboration relationship between countries or regions of the investigated 2839 papers. Analysis result reveals that collaboration exists among 101 countries or regions. There are totally 97 countries or regions with more than 1 citation, 61 with more than 100 citations, and 29 with more than 1000 citations. The USA is country which has the most citations, with a number of published articles of 413 and the number of citations of 23,856. China takes the second place with 550 published papers and 16,699 citations, followed by India with 354 published papers and 11,768 citations. Table 2 shows the collaboration network depicting the top 10 countries or regions with the most citations, and lists their published papers and total link strength.

**Table 2:** Top 10 countries or regions ranked by the number of citations in the collaboration network.

Country	Citation	Number of publication	Total link strength
USA	23,856	413	261
China	16,699	550	246
India	11,768	354	187
Germany	7202	156	109
South Korea	5492	126	109
Australia	5165	86	89
Italy	4723	112	76
England	4634	113	151
France	3928	102	104
Iran	3565	98	44

**Figure 3.**
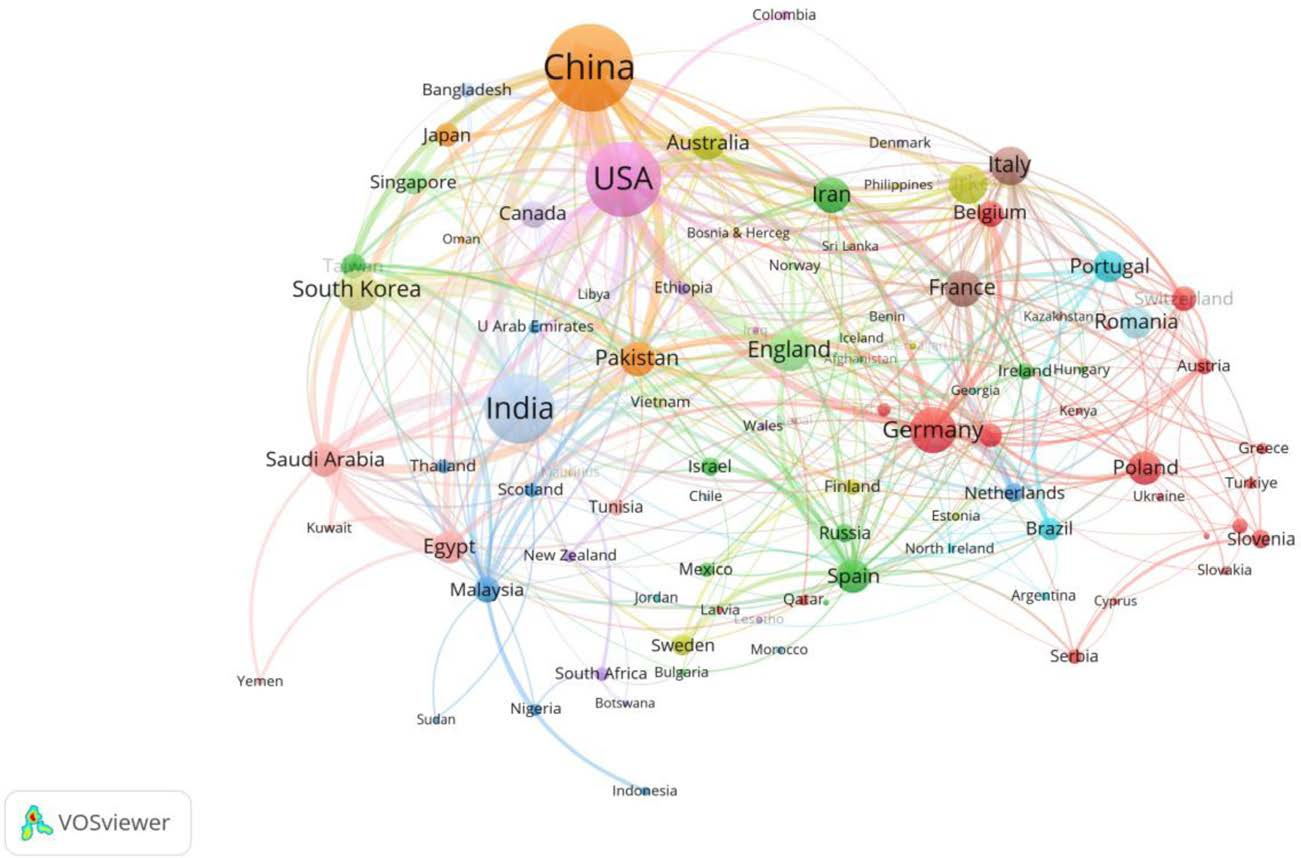
The mapping of countries or regions collaboration network in the research area of medical textiles.

A collaboration network is generated based on these 101 countries or regions as shown in Figure 3. In the figure, each node represents a country or region, and the more citations the country or region has, the larger of the node is. The total link strength represents the frequency of collaboration between a country/region and other countries/regions. As can be seen from the figure, China, the USA, India and several other countries or regions have relatively strong collaboration relationship.

### 3.4. Analysis of co-authorship between organizations

The co-authorship function of VOSviewer is used to analyze the collaboration relationship between organizations. From the retrieved 2839 papers there are totally 3151 organizations related to the research area of medical textiles. Among these organizations, 2892 have published papers cited more than once, 484 have published papers cited more than 100 times, and 25 have published papers cited more than 1000 times. With 46 published articles and 3117 citations, “Chinese Academy of Sciences” has the most citations, followed by “Harvard University” with 15 published articles and 3102 citations and “Indian Institute of Technology” with 24 published articles and 2888 citations. The collaboration network among the top 10 organizations with the most citations is listed in Table 3, which also lists their published articles and total link strength.

**Table 3:** The top 10 organizations ranked by the number of citations in the collaboration network.

Rank	Organization	Host country	Number of articles	Number of citation	Total link strength
1	Chinese Academy of Sciences	China	46	3117	33
2	Harvard University	USA	15	3102	11
3	Indian Institute of Technology	India	24	2888	3
4	National University of Singapore	Singapore	30	2264	9
5	University of Illinois	USA	6	1993	1
6	Ghent University	Belgium	33	1805	4
7	Donghua University	China	81	1572	26
8	Hong Kong Polytechnic University	China	59	1477	10
9	Georgia Institute of Technology	USA	17	1444	10
10	Tubingen University	Germany	2	1432	0

**Figure 4.**
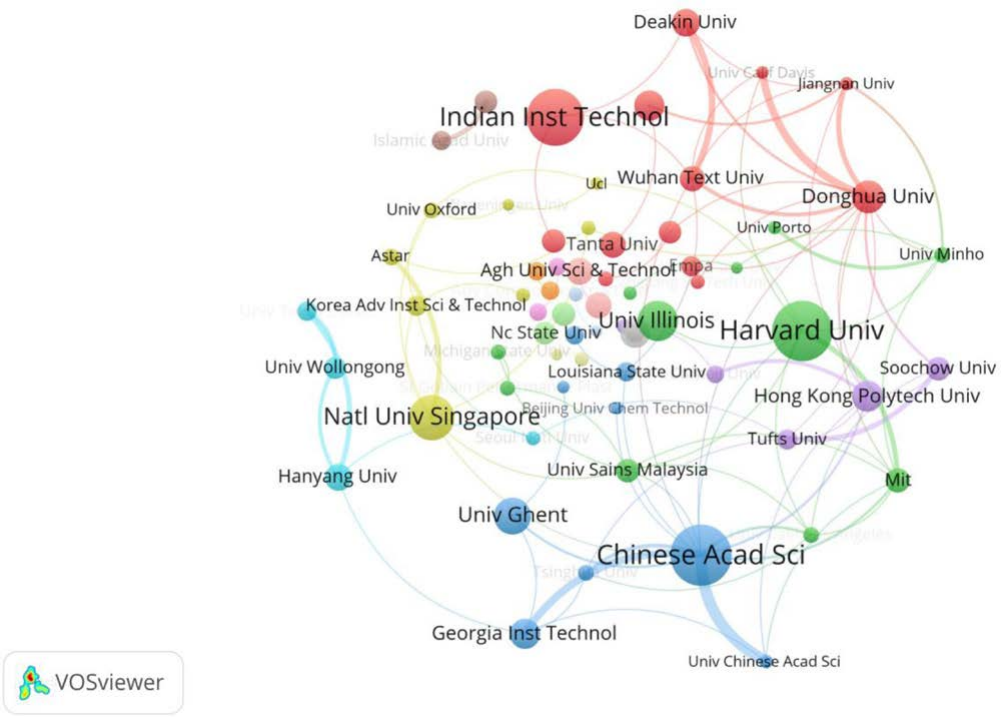
The mapping of organizations collaboration network in research area of medical textiles.

There are 73 organizations which have published papers cited over 500 times. The collaboration network among these organizations is depicted in Figure 4. Each node in the figure represents an organization, and the more citations affiliated with the organization, the larger of the node is. The total link strength stands for the frequency of the collaboration between an organizations and other organizations. Strong collaboration relations with other institutions can be found by “Chinese Academy of Sciences,” “Donghua University,” “Georgia Institute of Technology,” “Harvard University,” “Hong Kong Polytechnic University,” etc.

### 3.5. Analysis of co-authorship between authors

**Figure 5.**
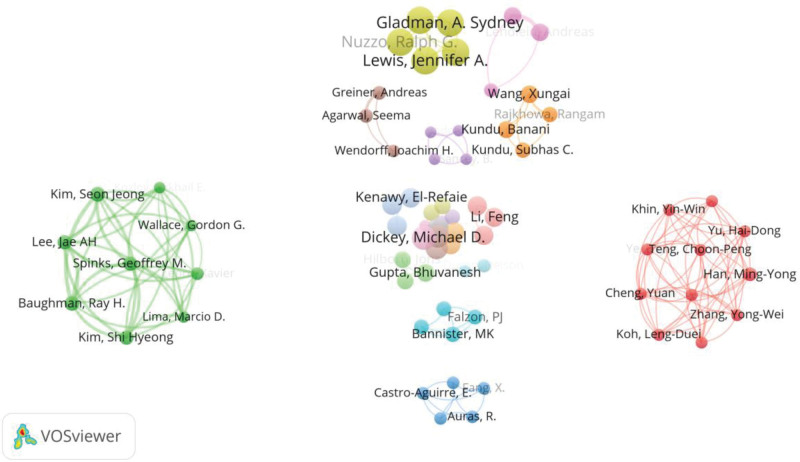
The mapping of authors’ collaboration network in the research area of medical textiles.

Using the co-authorship function of VOSviewer to analyze the collaboration relationship between the authors of 2839 articles. The result shows that 73 authors have been cited for more than 600 times. A collaboration network among these 73 authors is generated as shown in Figure 5. In the network, each node represents an author. The more citations an author has, the larger the node representing the author is. As can be seen from Figure 5, the 73 authors are categorized into 22 small groups, and the collaboration between the groups is not frequent.

These 2839 articles related to medical textiles are attributed to 11,146 authors, of which 10,846 have been cited more than once, 1047 have been cited more than 100 times, and 22 have been cited more than 1000 times. Table 4 lists the top 10 most cited authors and the number of citations. Among them, “Lewis Jennifer A.” from Harvard University is the most cited author. Her has published 2 articles that have been cited 1920 times in total. The total link strength between the authors is also listed, by which larger number refers to stronger collaboration of an author with other authors. Table 5 lists the top 10 authors ranked by the number of published articles. As can be seen form the table, “Montazer Majid” from “Amirkabir University of Technology” in Iran has published the most articles.

**Table 4:** Top 10 authors ranked by the number of citations in the collaboration network.

Rank	Author	Country	Organization	Number of articles	Number of citation	Total link strength
1	Lewis Jennifer A.	USA	Harvard University	2	1920	4
2	Gladman A. Sydney	USA	Harvard University	1	1868	4
3	Mahadevan L.	USA	Harvard University	1	1868	4
4	Matsumoto Elisabetta A.	USA	Harvard University	1	1868	4
5	Nuzzo Ralph G.	USA	University of Illinois	1	1868	4
6	Dickey Michael D.	USA	North Carolina State University	2	1590	1
7	Chen X.	Germany	University of Tubingen	1	1432	1
8	Schluesener H. J.	Germany	University of Tubingen	1	1432	1
9	Wang Zhong Lin	USA	Georgia Institute of Technology	7	1330	0
10	Broughton Roy	USA	Auburn University	1	1240	2

**Table 5:** Top 10 authors ranked by the number of published articles.

Rank	Author	Number of articles	Country	Organization
1	Montazer Majid	17	Iran	Amirkabir University of Technology
2	Rogier Hendrik	15	Belgium	Ghent University
3	Li Li	11	China	Hong Kong Polytechnic University
4	Ding Bin	11	China	Donghua University
5	Yu Jianyong	11	China	Donghua University
6	Lin Jia Horng	10	Germany	Feng Chia University
7	Cherif Chokri	10	Germany	Technische Universitat Dresden
8	Ramakrishna Seeram	10	Singapore	National University of Singapore
9	Sun Gang	10	USA	University of California Davis
10	Liu Xiangdong	9	China	Zhejiang Sci-Tech University

### 3.6. Source journals analysis

The investigated 2839 articles are published in 908 journals, of which 836 journals have been cited more than once, and 206 journals have been cited more than 100 times, and 18 journals have been cited more than 1000 times. Table 6 gives the top 10 journals with published articles, as well as the number of citations times, impact factors and H-index of the corresponding journals. The journal “Textile Research Journal” with 77 articles is the most cited journal, followed by the Journal of “Polymers” with 67 articles. Table 7 is a list of the top 10 journals with the number of co-citations, as well as the number of citations times, impact factors and H-index of the corresponding journals. The journal with the highest number of co-citations is “Carbohydrate Polymers,” with 3252 co-citations, followed by journal of “Advanced Materials” with 3233 co-citations.

**Table 6:** Top 10 journals in the research area of medical textiles ranked by the number of articles.

Rank	Journal title	Number of article	Number of citation	Impact factor (5-yr average)	H-index
1	Textile Research Journal	77	1056	2.3	74
2	Polymers	67	1162	5	53
3	Journal of Industrial Textiles	63	616	3.4	31
4	Journal of the Textile Institute	59	449	1.7	36
5	Fibers and Polymers	50	847	2.5	43
6	Sensors	47	1947	4.1	132
7	ACS Applied Materials & Interfaces	47	1917	9.6	169
8	Journal of Applied Polymer Science	45	886	2.9	149
9	Fibres & Textiles in Eastern Europe	37	432	0.9	33
10	International Journal of Biological Macromolecules	34	1710	7.8	101

**Table 7:** Top 10 journals in the research area of medical textiles ranked by the number of co-citations.

Rank	Journal title	Number of co-citation	Impact factor (5-yr average)	H-index
1	Carbohydrate Polymers	3252	7.536	172
2	Advanced Materials	3233	28.019	447
3	ACS Applied Materials & Interfaces	2843	9.6	169
4	Journal of Applied Polymer Science	2374	2.9	149
5	Biomaterials	2073	13.8	337
6	International Journal of Biological Macromolecules	1964	7.8	101
7	Advanced Functional Materials	1652	19.2	269
8	Textile Research Journal	1583	2.3	74
9	ACS Nano	1519	17.1	310
10	Polymer	1266	4.6	236

Co-citation analysis of journals can help readers to understand the distribution of important knowledge in a research field. Analyzing the co-citations and connections of journals can deliver readers an overview of how different areas of knowledge in these journals are clustered and distributed. This information can be extracted from the reference bibliography of these 2839 articles through using VOSviewer to conduct co-citation analysis. In this paper, 25,125 journals are analyzed. It shows that 278 journals are co-citation more than 100 times, and 17 journals were co-citation more than 1000 times.

**Figure 6.**
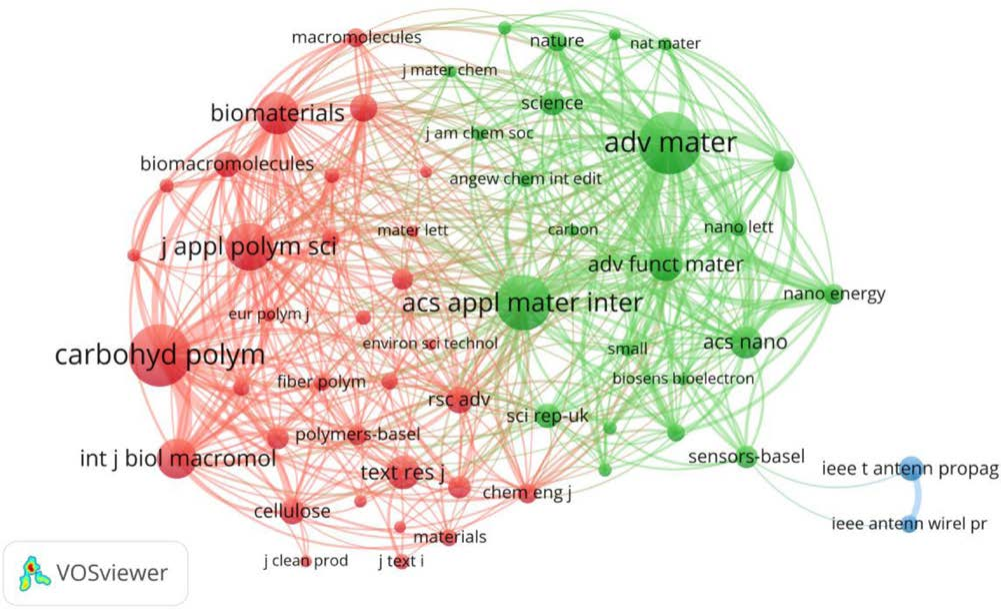
Co-citation clusters mapping of journals in the research area of medical textiles.

Figure 6 is the network diagram of co-citation of 57 journals with more than 500 times of co-citation. As shown in Figure 6, a node represents a journal, the size of the node reflects the number of co-citations of the journal. Larger the node refers to higher number of co-citations of the journal, and nodes of the same color are from the same cluster. The investigated 57 journals are divided into 3 clusters.

Cluster 1 consists of 32 red nodes. In this cluster, the journal with the highest number of co-citations is “Carbohydrate Polymers,” with a co-citations number of 3252. The 5-year average impact factor of the journal is 7.536, and the number of articles related to medical textiles is 19.

Cluster 2 consists of 23 green nodes. With a co-citations number of 3233, “Advanced Materials” is the journal with the highest number of co-citations in this cluster. Its 5-year average impact factor is 28.019, and the number of articles related to medical textiles is 16.

Cluster 3 consists of 2 blue nodes. In this cluster, the journal with the highest number of 1134 co-citations is “IEEE Antennas and Wireless Propagation Letters,” of which the 5-year average impact factor is 4.7, and the number of articles related to medical textiles is 17.

### 3.7. Citation analysis of articles

The citation relationship between scientific articles shows that scientific articles are not isolated but interrelated and expanding systems. The mutual citation of scientific article reflects the objective law of scientific development and the accumulation and continuation of scientific knowledge. It is a special form of retrieval system, which can trace the source forward and the development backward through the citation network. The citation frequency of scientific article is not balanced, and the sparsity of citation network density reflects the dispersion and concentration of citation distribution.^[11]^ Among the 2839 articles, 2543 are cited more than once, 208 are cited more than 100 times, and 6 articles are cited over 1000 times.

Table 8 lists the top 10 most cited articles in the research field of medical textiles. The first one is “Biomimetic 4D printing” published by “Gladman A. Sydney” in “Nature Materials” in 2016. It has been citated 1868 times. This article mainly focuses the usage of 4D biomimetic technology to print composite hydrogel structures with localized, anisotropic swelling behavior controlled by the arrangement of cellulose fibrils along a prescribed 4D printing path.^[27]^ The second one is “Nanosilver: A nanoproduct in medical application” published by “Chen X.” in “Toxicology Letters” in 2008, and it has been cited 1432 times. This article discusses the main issues for the medical use of nanosilver and related nanomaterials.^[10]^ On the third place is “The chemistry and applications of antimicrobial polymers: A state-of-the-art review,” which is published by “Kenawy El-Refaie” in “Biomacromolecules” in 2007 and has been cited 1240 times. The research of antimicrobial polymer development is a great challenge to academia and industry, this article mainly discusses the requirements of antimicrobial polymers, factors affecting antimicrobial activity, methods of synthesizing antimicrobial polymers, applications of antimicrobial polymers in major fields, future prospect.^[30]^

**Table 8:** The top 10 most cited articles.

Rank	Title	The first author	Year	Number of citation	Source journal
1	Biomimetic 4D printing	Gladman A. Sydney	2016	1868	Nature Materials
2	Nanosilver: a nanoproduct in medical application	Chen X.	2008	1432	Toxicology Letters
3	The chemistry and applications of antimicrobial polymers: a state-of-the-art review	Kenawy El-Refaie	2007	1240	Biomacromolecules
4	Phase change materials for thermal energy storage	Pielichowska Kinga	2014	1238	Progress in Materials Science
5	Progress in flexible lithium batteries and future prospects	Zhou GuangMin	2014	1192	Energy & Environmental Science
6	Stretchable and soft electronics using liquid metals	Dickey Michael D.	2017	1018	Advanced Materials
7	Poly (lactic acid) fiber: an overview	Gupta Bhuvanesh.	2007	960	Progress in Polymer Science
8	Industrial and biotechnological applications of laccases: a review	Couto Susana Rodriguez	2006	949	Biotechnology Advances
9	Silk fibroin biomaterials for tissue regenerations	Kundu Banani	2013	874	Advanced Drug Delivery Reviews
10	A review of stimuli-responsive shape memory polymer composites	Meng Harper	2013	859	Polymer

### 3.8. References co-citation analysis

The concept of article co-citation analysis is proposed by Henry Small in 1973. Co-citation analysis refers to that 2 articles appear together in the reference list of a third articles, and these 2 articles form a co-citation relationship. Through mining process of the co-citation relationship of articles in the spatial data collection of articles, it can be considered as the co-citation analysis of articles.^[31]^ Literature co-citation analysis can be used to measure the similarity of 2 articles. It can also be used to study the internal relationship of scientific literatures and describe the dynamic structure of scientific development.

These 2,839 articles have 142,378 references in total, among which 425 references have been co-citation over 10 times, and 10 references are co-citation more than 35 times. Table 9 lists the first author, publication year, co-citation times, source journal of these 10 references, respectively. The most co-citation references are “Stoppa Matteo” published in “Sensors” in 2014 with the title “Wearable electronics and smart textiles: a critical review,” has been co-citation 66 times. This review highlights recent advances in the field of smart textiles, especially regarding materials for smart textiles and their manufacturing processes.^[32]^

**Table 9:** The top 10 most co-cited references in the research area of medical textiles.

Rank	Title	The first author	Year	Number of co-citation	Source journal
1	Wearable electronics and smart textiles: a critical review	Stoppa Matteo	2014	66	Sensors
2	Recent advances in antimicrobial treatments of textiles	Gao Yuan	2008	64	Textile Research Journal
3	Silk-based biomaterials	Altman GH	2003	48	Biomaterials
4	Dual-band wearable textile antenna on an EBG substrate	Zhu ShaoZhen	2009	48	IEEE Transactions on Antennas and Propagation
5	Structures of novel antimicrobial agents for textiles – a review	Simoncic Barbara	2010	38	Textile Research Journal
6	Fiber-based wearable electronics: a review of materials, fabrication, devices, and applications	Zeng Wei	2014	38	Advanced Materials
7	A review on the application of inorganic nano-structured materials in the modification of textiles: focus on anti-microbial properties	Dastjerdi Roya	2010	36	Colloids and Surfaces B-Biointerfaces
8	Fully integrated wearable sensor arrays for multiplexed in situ perspiration analysis	Gao Wei	2016	35	Nature
9	Low-profile dual-band textile antenna with artificial magnetic conductor plane	Yan Sen	2014	34	IEEE Transactions on Antennas and Propagation
10	Silver nanoparticles as a new generation of antimicrobials	Rai Mahendra	2009	34	Biotechnology Advances

**Figure 7.**
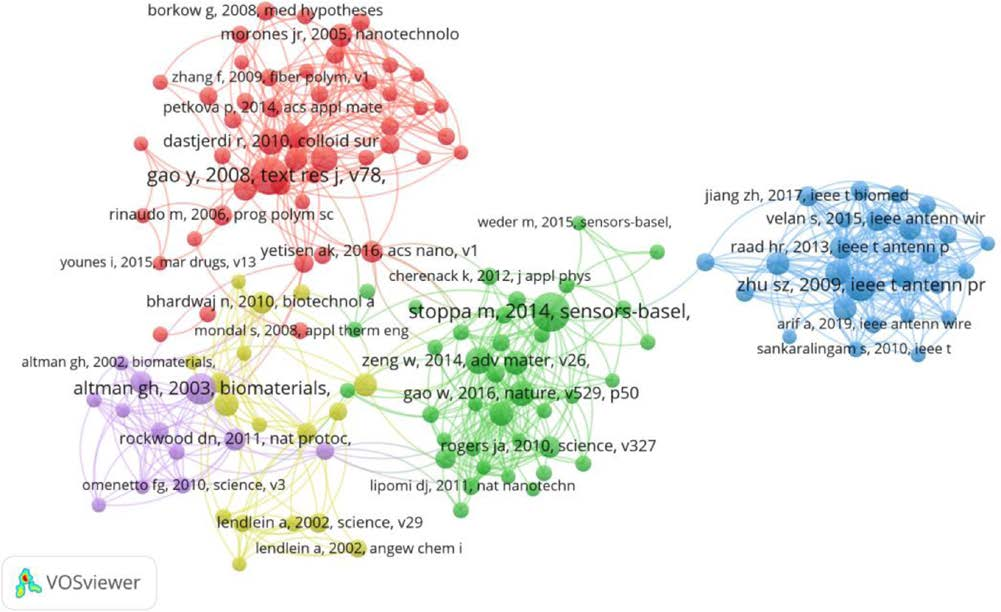
The mapping of co-citation cluster of references.

A co-citation analysis is performed on 142 references with over 15 co-citations using VOSviewer, and a mapping of co-citation cluster of references is generated in Figure 7. As can be seen from Figure 7, the references are divided into 5 clusters. Different clusters are represented by nodes in different colors, and each node represents a reference. The larger the node is, the more co-citations the reference has.

Cluster 1 in the Figure 7 is in red. It includes 48 references, in which the most co-citation one is published by “Gao Yuan” in the journal “Textile Research Journal” in 2008, the number of co-citations is 64.

Cluster 2 is in green containing 39 references. In this cluster, the most co-citation reference is published by “Stoppa Matteo” in the journal “Sensors” in 2014, and it is co-citation for 66 times.

Cluster 3 is in blue with 26 references, in which the most co-citation one is published by “Zhu Shaozhen” in the journal “IEEE Transactions on Antennas and Propagation” in 2009, with a number of co-citations of 48.

Cluster 4 is in yellow. It has 17 references, the most co-citation one is published by “Huang ZM” in the journal “Composites Science and Technology” in 2003, and it is been co-citation 31 times.

Cluster 5 is in purple with 12 references, its most co-citation reference is published by “Altman GH” in the journal “Biomaterials” in 2003, with a number of co-citations of 48.

## 4. Research hotspots and trends

### 4.1. Research hotspots

In 1983, Michelle Cullen proposed a concept of co-word analysis, of which the main method is to count the strength of association by the number of co-occurrences between different subject words, keywords, etc. The specific method is to count the co-occurrence frequency of subject words or keywords in a group of texts, to establish the co-occurrence matrix, to conduct distance statistics and network analysis.^[33]^ Co-word analysis is to trace the development of science by finding the connection between various disciplines in a research field through the common occurrence frequency of words or phrases.^[34]^

There are totally 7684 author keywords and 6151 keywords plus in these retrieved 2839 articles. After deduplication, the total number of keywords is reduced to 12,665. Keywords plus is a keyword supplemented by the core collection of Web of Science™ provider according to the content of the articles. Using VOSviewer to analyze the co-occurrence of all keywords reveals that, there are 47 keywords with over 50 co-occurrence times, and 22 keywords with over 100 co-occurrence times. Table 10 lists the top 20 keywords with their co-occurrence times. “textile” have the highest number of co-occurrences, up to 360 times, follow by “fiber” of 214 time and “silver nanoparticle” with 205 times.

**Table 10:** The top 20 keywords by co-occurrence times.

Rank	Keyword	Count	Rank	Keyword	Count
1	textile	360	11	design	143
2	fiber	214	12	sensor	129
3	silver nanoparticle	205	13	surface	127
4	nanoparticles	188	14	fabrication	122
5	antibacterial	181	15	film	120
6	medical textile	165	16	polymer	118
7	antibacterial activity	164	17	performance	115
8	chitosan	160	18	fabrics	115
9	mechanical-properties	151	19	cotton	111
10	composites	149	20	cotton fabrics	111

**Figure 8.**
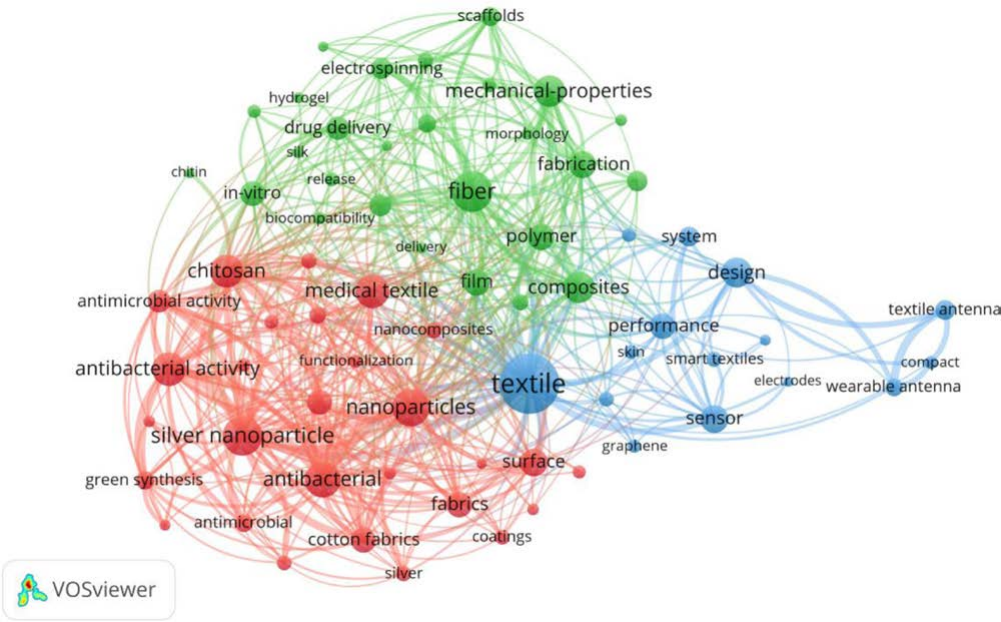
Co-occurrence cluster mapping of keywords (by VOSviewer).

**Figure 9.**
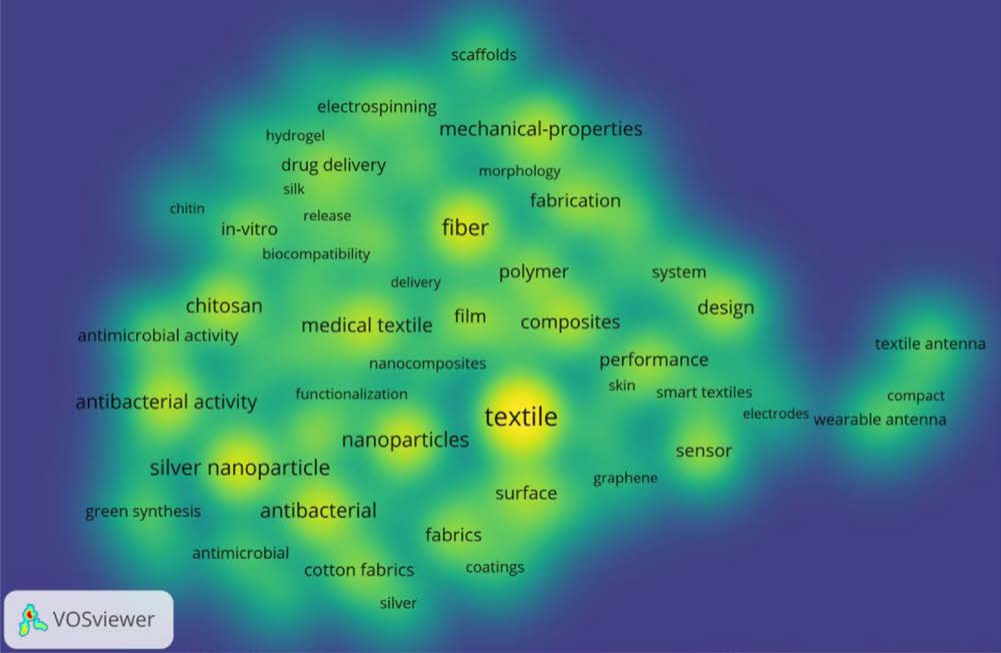
Co-occurrence cluster density visualization of keywords (by VOSviewer).

Based on 70 keywords with over 35 co-occurrences times, a cluster map of keyword co-occurrence distribution is generated as shown in Figure 8. In Figure 8, 1 node represents a keyword, all nodes in the same color belong to the same cluster. The larger a node is, the higher the co-occurrence times it refers to. As can be seen from Figure 8, these 70 keywords are divided into 3 clusters. Figure 9 visualizes the co-occurrence cluster density of keywords.

Cluster 1 consists of 28 red nodes. The top 3 keywords with the most co-occurrences are “silver nanoparticle,” “nanoparticles,” and “antibacterial” with co-occurrence times of 205, 188, and 181, respectively. Important keywords in this cluster are “silver nanoparticles,” “antibacterial activity,” “chitosan,” etc. This cluster mainly revolves around the application of nanomaterials and biopolymers such as chitosan and their derivatives in medical textiles. “Chen X.” (2008) pointed out that nanosilver is used in various textile coatings, as well as some implant coatings due to its strong antibacterial activity. The use of nanosilver in medicine is becoming more common, and due to the increased exposure, the toxicological and environmental issues of nanosilver need to be explored.^[10]^ “Jung Kyung-Hye” (2007) explored the introduction of antibacterial activity and biocompatibility into the surface of polyethylene terephthalate textiles using electrospinning nanofiber deposition method.^[12]^ “Krishnamoorthy Karthikeyan” (2012) reported surface modification of cotton fabrics using graphene oxide nanostructures, and graphene oxide-coated cotton fabrics were more toxic to Gram-positive bacteria.^[13]^ “El-Rafie M. H.” (2014) discusses an ecological and feasible method for coating cotton fabrics with silver nanoparticless (AgNPs), regardless of the concentration of AgNPs used, without washing, with consistently high bactericidal ability.^[14]^ “Renault F.” (2009),^[35]^ “Hamed Imen” (2016),^[36]^ and “Senel S” (2004)^[37]^ all mentioned that chitosan can be used in medical textiles.

Cluster 2 includes 27 green nodes. In this cluster, the top 3 keywords with the most co-occurrences are “fiber,” “mechanical-properties,” “composites,” and the co-occurrence times are 214, 151, and 149, respectively. Other important keywords in this cluster include “electrospinning,” “scaffolds,” “drug delivery,” etc. This cluster centers on electrospinning nanoscale polymer fibers for medical applications such as in vitro and in vivo drug delivery. “Subtirica Adriana-Ioana” (2018) and “Cheng Huiling” (2018) mainly introduced PEO and PVA nanofibers obtained by electrospinning process are, and the biocompatible and biodegradable polymers of such fibers are made into microfiber webs. They possess unique properties such as high surface area to volume ratio, small pore size, high porosity, and incorporation of therapeutic compounds into electrospun nanofibers.^[15]^ “Cheng Huiling” (2018) mainly introduced the biomedical applications of electrospun fibrous materials, controlled drug release from electrospun fibrous materials, and recent advances in devices to produce complex electrospun fibrous materials are highlighted.^[9]^ Introduced a way to combine a substrate-free electrospinning process with textile technology to develop a new collector design that provides a pressure-driven localized lint structure in free space from which continuous the advantage of this textile is that it can be loaded with drugs/dyes and developed into a core-sheath structure with greater functionality.^[38]^

Cluster 3 contains 15 blue nodes. The top 3 keywords with the most co-occurrences in this cluster are “textile,” “design,” “sensor,” with the co-occurrence of 360, 143, and 129 times, respectively. Further important keywords in the cluster include “performance,” “textile antenna,” “smart textiles,” etc. This cluster is mainly related to the medical applications of smart textiles such as wearable sensors and wearable antennas. “Carpi F” (2005) introduced early concepts and recent developments in electroactive polymer-based sensors, actuators, electronic components, and power sources, which become wearable devices for smart textiles.^[39]^ “Lin Zhiming” (2018) introduced a pressure-sensitive, large-scale, washable smart textile for bed sheets based on triboelectric nanogenerator arrays is reported for real-time and self-powered monitoring of sleep behavior.^[40]^ “Li Yi” (2019) reviewed the theoretical basis and progress of textile biomedical engineering in recent years.^[5]^

### 4.2. Evolution of research directions and research fronts

Using the software CiteSpace to analyze the co-occurrence of keywords can be used to determine the research hotspots in a research field. Research hotspots refer to one or more topics of common concern among scholars, which have strong temporal characteristics. Co-occurrence cluster of keywords can also be used to reveal the current research directions in a research field, and this can help researchers understand the evolution of the research hotspots as well as the future research trends.

**Figure 10.**
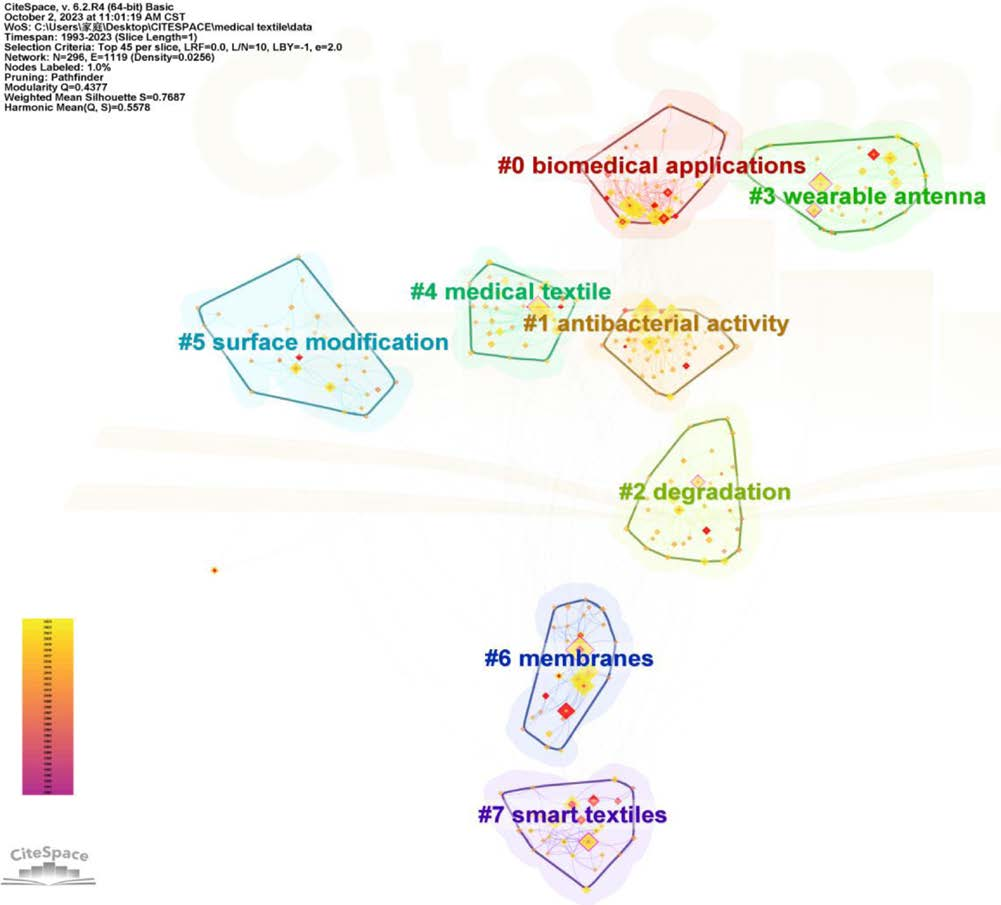
Co-occurrence cluster mapping of keywords.

In this paper, CiteSpace is used for co-occurrence clustering of all author keywords and keywords plus. There are 8 main clusters generated, among which the information of the 8 clusters with the largest number of clustering words is shown in Figure 10 and Table 11. The number of keywords, silhouette, and extracted LSI algorithm labels of these 8 clusters are listed in Table 11. Silhouette is used to evaluate the validity of clustering. Silhouette value greater than 0.5 indicates that a clustering is reasonable, while silhouette value over 0.7 suggests a convincing clustering. At the same time, the betweenness centrality of each keyword in the co-occurrence cluster network is calculated. Betweenness centrality is a measure of the importance of nodes in a network. The betweenness centrality of keywords is used to discover and measure the importance of keywords. Keywords with high betweenness centrality are usually the key hubs connecting 2 different keywords. By selecting top 100 levels of most co-occurrence keywords from each slice, top 10% of most co-occurrence keywords per slice, and top 45 maximum number, CiteSpace was used to calculate the author keywords and keywords plus betweenness centrality. There are 7 keywords with betweenness centrality greater than 0.1, which are textile (0.18), design (0.17), medical textile (0.13), system (0.13), film (0.12), surface (0.1), and behavior (0.1).

**Table 11:** Keywords co-occurrence cluster information.

Cluster	Size	Silhouette	LSI	Top 10 co-occurrence keywords
#0	43	0.831	Biomedical applications	Mechanical property (182); fabrication (107); in vitro (102); drug delivery (97); biomedical applications (85); scaffolds (72); nanofibers (38); controlled release (25); silk fibroin (25); removal (16)
#1	40	0.765	Antibacterial activity	Silver nanoparticle (204); antibacterial activity (167); cotton fabrics (108); antimicrobial activity (95); green synthesis (71); adsorption (37); in situ synthesis (26); toxicity (26); cellulose (21); zno nanoparticles (18)
#2	36	0.646	Degradation	Fabrics (101); behavior (67); degradation (45); water (23); morphology (18); extraction (15); strength (7); extract (7); photocatalytic activity (7); particles (6)
#3	32	0.823	Wearable antenna	Design (135); textile antenna (77); system (76); wearable antenna (66); compact (39); strain (8); biomedical monitoring (8); arrays (8); slot antenna (7); oxide (6)
#4	32	0.723	Medical textile	Medical textile (159); composites (116); chitosan (82); cotton (54); temperature (37); nanocomposites (28); graphene oxide (23); delivery (22); functionalization (12); cross linking (11)
#5	29	0.809	Surface modification	Polymer (82); surface modification (55); coatings (13); polymerization (7); silver (7); antioxidant (6); light (6); networks (5); protein (4); bacteria (4)
#6	28	0.877	Membranes	Textile (199); fiber (172); nanoparticles (146); surface (123); antibacterial (82); escherichia coli (31); membranes (23); deposition (14); staphylococcus aureus (5); optimization (5)
#7	27	0.761	Smart textiles	Film (111); performance (110); sensor (87); smart textiles (46); carbon nanotubes (41); skin (25); wearable electronics (22); transparent (17); wearable sensors (14); hydrogels (11)

**Figure 11.**
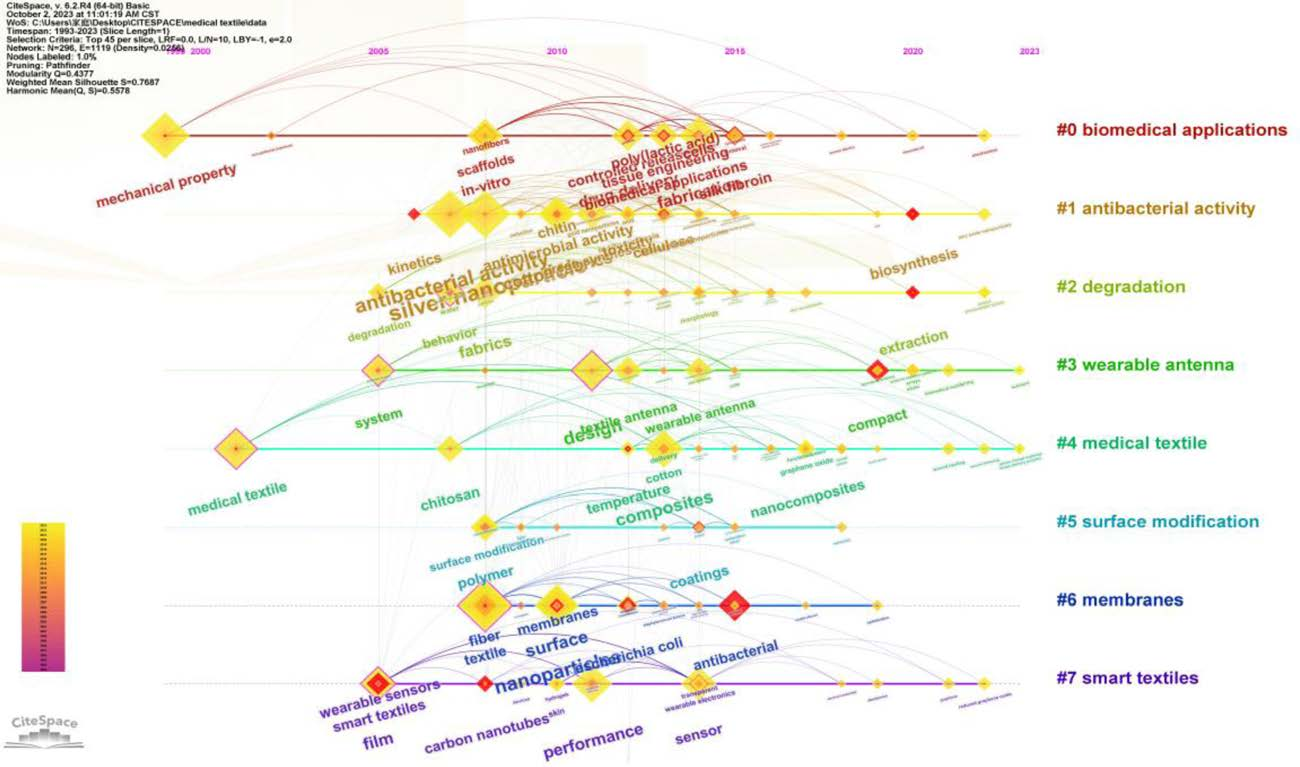
The timeline view of keywords co-occurrence clusters.

The keyword co-occurrence cluster mapping can be switched into timeline view. In the timeline view of the keyword clustering, all keywords of the same cluster are located on the same horizontal line. The time axis of keywords appearance is set at the top of the view. The number of keywords in each cluster can be obtained in the timeline view. Larger number of keywords in a cluster indicates that the cluster is more important in the field. Meanwhile, the time span as well as its rise and fall process of each keyword in the cluster can be plotted. To further explore the time characteristics of a research field reflected by the cluster, the important keywords in each cluster can be measured by burst detection and betweenness centrality index. In the timeline view, the horizontal axis is the timeline, and the vertical axis stands for the clustering of different keywords. Each node represents a different burst detection keyword, and larger node refers to greater burst detection value of the keyword. Figure 11 is a timeline view of the clustering of keywords, and 1 line in the figure represents a research direction of medical textiles evolving with time.

Timeline 0# contains 43 keywords, the keywords with burst detection are “mechanical property” (4.29, 1999), “controlled release” (6.39, 2012), “poly (lactic acid)” (3.65, 2013), “cells” (4.07, 2014), “biomedical applications” (5.5, 2017), “tissue engineering” (3.67, 2019), “in vitro” (6.08, 2020) and “silk fibroin” (4.2, 2021). The numbers in parentheses refer to the burst detection values and the burst begin year, respectively. The keywords with more co-occurrence times are “mechanical property” (182, 1999), “fabrication” (107, 2014), “in vitro” (102, 2008), “drug delivery” (97, 2012), etc. Here, the numbers in parentheses are the co-occurrence frequency and the year of occurrence, respectively. This cluster is mainly affiliated with the superior mechanical properties of silk fibroin that can be composite with micro-nano processing to fabricate a drug delivery system. This can realize the combined delivery of a variety of drugs and the controlled release of drug molecules.

Timeline 1# contains 40 keywords, the keywords with burst detection are “kinetics” (4.09, 2006), “chitin” (3.65, 2010), “cellulose” (5.77, 2013), “toxicity” (3.49, 2018), and “biosynthesis” (6.74, 2020). The numbers in parentheses refer to the burst detection values and the burst begin year, respectively. The keywords with more co-occurrence times are “silver nanoparticle” (204, 2008), “antibacterial activity” (167, 2007), “cotton fabrics” (108, 2010), “antimicrobial activity” (95, 2010), etc. Here, the numbers in parentheses are the co-occurrence frequency and the year of occurrence, respectively. This cluster is mainly related to the efficacy of medical textiles processing such as the addition of nanocoating to achieve antimicrobial function of textiles.

Timeline 2# covers 36 keywords. The burst detection keywords in this cluster include “extraction” (7.22, 2020). The keywords with more co-occurrence times include “fabrics” (101, 2008), “behavior” (67, 2007), “degradation” (45, 2005), and “water” (23, 2007), etc. This cluster mainly focuses on methods used for degrading pollutants in waste water. These pollutants are related to coatings and polymers in medical textiles.

Timeline 3# covers 32 keywords. The burst detection keywords in this cluster include “compact” (6.1, 2020). The keywords with more co-occurrence times include “design” (135, 2011), “textile antenna” (77, 2012), “system” (76, 2005), etc. This cluster mainly focuses on the application of textile antennas, wearable antennas, and other compact systems combined with flexible textiles in the medical field.

Timeline 4# includes 32 keywords. In this cluster, the burst detection keywords involve “medical textile” (6.6, 2001), “cross linking” (4.46, 2013), “nanocomposites” (4.62, 2017) and “temperature” (3.92, 2017). The keywords with more co-occurrence times are “composites” (116, 2013), “chitosan” (82, 2007), “cotton” (54, 2013), “graphene oxide” (23, 2017), etc. This cluster revolves around nanocomposites such as chitosan used to manufacture cotton fabrics as composite antimicrobial coating. Such nanocomposites can significantly improve the performance of textiles in specific biomedical applications. They can be utilized for a variety of purposes in human tissues such as immune stimulation, drug delivery, healing of wound, and blood coagulation.

Timeline 5# includes 29 keywords. In this cluster, the burst detection keyword is “coatings” (5.76, 2014). The keywords with more co-occurrence times are “polymer” (82, 2008), “surface modification” (55, 2008), etc. This cluster is mainly related to biofilm through coating on textile with polymer or antioxidants, so that the textile surface can be modification to achieve the function of antibacterial or drug release.

Timeline 6# contains 28 keywords, in which the burst detection keywords are “fiber” (3.54, 2008), “membranes” (4.14, 2010), “deposition” (4.98, 2010), “textile” (5.55,2017), “Escherichia coli” (4.46, 2019) and “antibacterial” (5.84, 2021). The keywords with more co-occurrence times include “nanoparticles” (146, 2010), “surface” (123, 2010), etc. This cluster primarily related to composite antimicrobial coatings on cotton fabrics using biopolymers such as nanoparticles. These nanoparticles form an overlaying membrane on the textile surface, it can significantly improve the performance of textiles in specific biomedical applications, such as preventing the transfer of pathogens like Klebsiella pneumoniae, Escherichia coli, Staphylococcus aureus etc. and reducing the incidence rate of infection.

Timeline 7# covers 27 keywords, in which the burst detection keywords are “wearable sensors” (3.67, 2017), “sensor” (3.93, 2018), “smart textiles” (6.81, 2021), and “carbon nanotubes” (3.94, 2021). The keywords with more co-occurrence times are “film” (111, 2005), “performance” (110, 2011), “skin” (25, 2010), “wearable electronics” (22, 2014), etc. This cluster represents the application of smart textiles in medical applications, where various devices are implanted into yarn or cotton fabrics, such as graphene films combined with clothing materials, and wearable electronic sensors embedded into textile fabrics or clothing for biomedical monitoring purposes.

**Figure 12.**
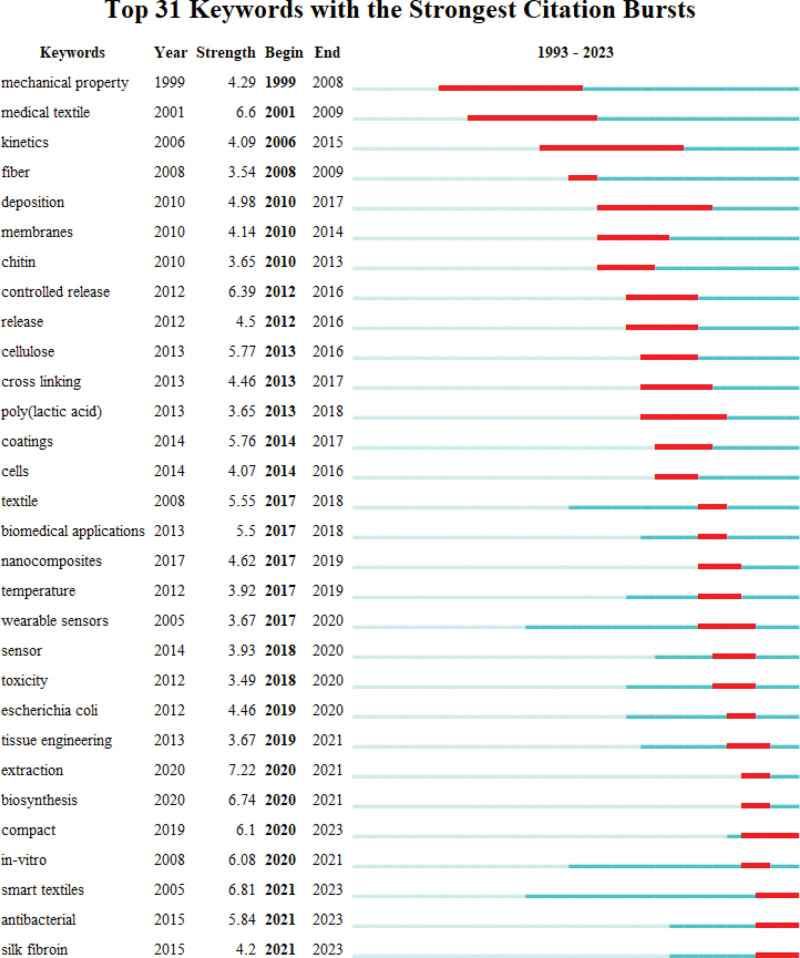
Thirty-one keywords with the strongest citation burst.

Research hotspots can be considered as one or more topics that researchers in a certain field are interested in. These topics have strong temporal characteristics.^[11]^ Burst detection can be used to detect a keyword in a short period of time when the frequency of its citation surge and the keyword becomes a research hotspot. There are totally 31 burst detection keywords in the field of. The emergence and evolution of research hotspots related to medical textiles can be roughly understood through the emergence time and end time of these burst detection keywords. Figure 12 lists the burst intensity, year of occurrence, begin time, and end time of the 31 burst keywords in the research field of medical textiles. In the long bud stage (1979–2002), “mechanical property” and “medical textile” are burst detection keywords, with strength value of 4.29 and 6.6, their appeared from 1999 to 2009. In the leap stage (2003–2013), have 10 burst detection keywords, the highest intensity burst detection keywords include “controlled release,” “cellulose” and “deposition,” with strength values of 6.39, 5.77, and 4.98, respectively. Their co-occurrence times are 2012–2016, 2013–2016, and 2010–2017, respectively. The remaining 19 burst detection keywords appear in the high-speed development stage, which starts from 2020 till now, with the highest intensity of “extraction” and “smart textiles,” whose burst detection values are 7.22 and 6.81, respectively.

## 5. Conclusions

From the perspective of bibliometrics, a systematic visual analysis of researches in the field of medical textiles is conducted in this paper. A total number of 2839 articles with a publication time span from 1979 to 2023 were retrieved from core collection of Web of Science™. Since 2014, the research on medical textiles has entered a high-speed development stage, with a significant increase in both the number of articles and the number of citations. It involves 105 research areas, among which the main research areas include material science, engineering, chemistry, polymer science. The most cited article is “Biomimetic 4D printing” published in “Nature Materials” in 2016 by “Gladman A. Sydney” from “Harvard University” with 1868 citations.

The software VOSviewer was used to analyze the co-authorship, co-citation, and co-occurrence of these 2839 articles. It is found that the USA, China, and India are outstanding in both the number of publications and the number of citations. Meanwhile, they have a strong collaboration relationship with other countries. The research organizations from these 3 countries are also strong. Ranked by the number of citations, the leading research organizations are “Harvard University,” “Indian Institute of Technology,” and “Chinese Academy of Sciences.” Regarding the terms of number of publications, “Hong Kong Polytechnic University,” “Chinese Academy of Sciences,” and “Donghua University” are the top 3 organizations. However, the collaboration between these organizations are not active. The authors can be divided into smaller groups, between which the collaboration is neither active. The most prolific author is “Montazer Majid” from “Amirkabir University of Technology” with 17 articles. The most cited is “Lewis Jennifer A.” from “Harvard University” with 1920 citations. The co-citation analysis of the journals indicated that “Textile Research Journal,” “Journal of Industrial Textiles,” and “Polymers” have published most articles on medical textiles, while “Carbohydrate Polymers,” “Advanced Materials,” “ACS Applied Materials & Interfaces” have most co-citations. The co-citation analysis of the references of these 2839 articles revealed that the article “Wearable electronics and smart textiles: a critical review” published in “Sensors” by “Stoppa Matteo” in 2014 is most frequently co-citation. Through the co-occurrence analysis of keywords, it was found out that the greatest number of co-occurrence keywords are “textile,” “silver nanoparticles,” “fiber,” etc.

A co-occurrence cluster analysis of keywords was also performed by using CiteSpace in this paper. The evolution process of the research hotspots and the main research directions in the field of medical textiles are analyzed. Generally, this paper presents a general, comprehensive, and clear analysis of medical textiles research from the perspective of bibliometrics. Although VOSviewer and CiteSpace have their own limitations, the analysis results are objective, stable and reliable. This paper also has its limitations. First, the scope of this study is limited to English articles collected from the core collection of Web of Science™. Second, although the author has analyzed the research hotspots and directions, the analysis and interpretation of the results is relatively subjective.

## Author contributions

**Conceptualization:** Zhiqun Liu.

**Formal analysis:** Zhiqun Liu.

**Investigation:** Fangping Yin, Nan Ruan.

**Software:** Zongzhan Gao.

**Supervision:** Zhiqun Liu.

**Validation:** Fangping Yin, Nan Ruan.

**Writing – original draft:** Zhiqun Liu.

**Writing – review & editing:** Zhiqun Liu.
